# Multifocal cellulitis and polymicrobial infection in a patient with a perforated duodenal ulcer: a case report and literature review

**DOI:** 10.1097/RC9.0000000000000883

**Published:** 2026-08-19

**Authors:** Tan Trong Tran, Duy Quang Ngo

**Affiliations:** Department of Gastrointestinal Surgery, Nhan dan Gia Dinh Hospital, Ho Chi Minh City, Viet Nam

**Keywords:** cellulitis, painkiller, perforated duodenal ulcer, Salmonella

## Abstract

**Introduction:**

Perforated peptic ulcer (PPU) is a critical surgical emergency associated with high mortality rates, typically requiring immediate surgical intervention. *Helicobacter pylori* infection and misuse of nonsteroidal anti-inflammatory drugs are common causes. Salmonella, a Gram-negative bacterium, is usually acquired through foodborne or fecal-oral exposure and most commonly causes gastroenteritis, bacteremia, or focal infection. Cellulitis, a bacterial infection involving the deep dermis and subcutaneous tissue, remains primarily a clinical diagnosis because no definitive gold-standard diagnostic test is currently available.

**Case presentation:**

A 41-year-old man, who had a long-term history of taking unidentified analgesics for chronic left groin pain, presented to the hospital with fever, diffuse abdominal pain, and both legs were red and painful. Initial evaluation suggested a perforated peptic ulcer and cellulitis of both legs and scalp, and emergency exploratory laparotomy and abscess drainage were performed simultaneously, revealing a duodenal perforation. Notably, blood cultures were positive for *Salmonella*, while wound cultures from the scalp and leg abscesses identified *Staphylococcus aureus*. The patient received intravenous antibiotics and nutritional support, leading to a full recovery.

**Discussion:**

The relationship between *Salmonella bacteremia*, duodenal perforation, and analgesic use cannot be proven from a single case. These findings are best interpreted as an unusual association in which several factors may have contributed to disease severity.

**Conclusion:**

This case highlights the importance of cautious etiologic interpretation, early source control, culture-directed antibiotics, and individualized postoperative nutritional management in complex perforated ulcer disease with multifocal infection.

## Introduction

Peptic ulcer disease affects approximately 4 million people annually and has a lifetime prevalence of 5–10%; approximately, 5% of affected patients may progress to perforation^[^1^]^. It often occurs in individuals between 25 and 50 years of age and in those involved in heavy manual labor, such as workers and farmers. In Vietnam, a report by Tran Thien Trung found that most patients were farmers (55.8%) and workers (8.1%)^[^2^]^. In April 1982, two Australian physicians, Warren and Marshall, discovered that *Helicobacter pylori* is the main cause of peptic ulcers and stomach cancer^[^3^]^. Perforated duodenal ulcers require early recognition, hemodynamic resuscitation, broad-spectrum antimicrobial therapy, and definitive source control. Operative strategies depend on patient physiology, perforation size, contamination, available tissue, and institutional practice^[^1,4^]^.

HIGHLIGHTS

- A perforated duodenal ulcer may present with multifocal polymicrobial infection.
- Occupational and environmental exposures should be considered in cases of unusual infections.
- Early surgery, targeted antibiotics, and nutritional support lead to good outcomes.

*Salmonella* live in the intestines of humans and animals. There are many ways to get infected, including eating contaminated food, drinking contaminated water, and touching animals^[^5^]^. Intestinal perforation related to enteric fever most commonly involves the terminal ileum, and gastroduodenal perforation associated with Salmonella infection has been reported only rarely^[^6^]^. We highlight the rarity of Salmonella infection associated with duodenal perforation, a clinical entity that is scarcely reported in the literature. In addition, this case provides important insights into the management of a complex fistula in this setting. We also describe unique patient-related factors, including occupational considerations, which may have contributed to disease presentation and management challenges. These aspects underscore the novelty and clinical relevance of our report and clarify why this case is important for readers.

## Case description

This case report has been prepared in line with the SCARE (Surgical CAse REport) criteria^[^7^]^. A 41-year-old male patient came to our Emergency Department (ED) with a 1-day history of acute abdominal pain, high-grade fever, and bilateral lower extremity edema, along with a 2-month history of chronic left groin pain. He had been taking unspecified analgesics without medical supervision. The type, dose, and frequency of the analgesics could not be reliably determined, but his condition worsened and he could not walk normally. After that, he developed progressive abdominal pain, mainly in the central abdomen, and he also developed a high fever. The patient’s past medical history was unremarkable. He worked as a sanitation worker and had no known history of diabetes mellitus, obesity, alcohol misuse, smoking, malignancy, or immunosuppressive therapy.

On admission, he was alert and responsive. His vital signs were as follows: blood pressure of 130/80 mmHg, heart rate of 140 bpm, temperature of 40 °C, respiratory rate of 22 breaths per minute, and oxygen saturation of 98% on room air. On abdominal palpation, there was generalized distension with severe tenderness in the epigastrium and periumbilical region, raising concern for peritonitis. On auscultation, low-pitched bowel sounds were present, suggestive of ileus.

**Figure 1.**
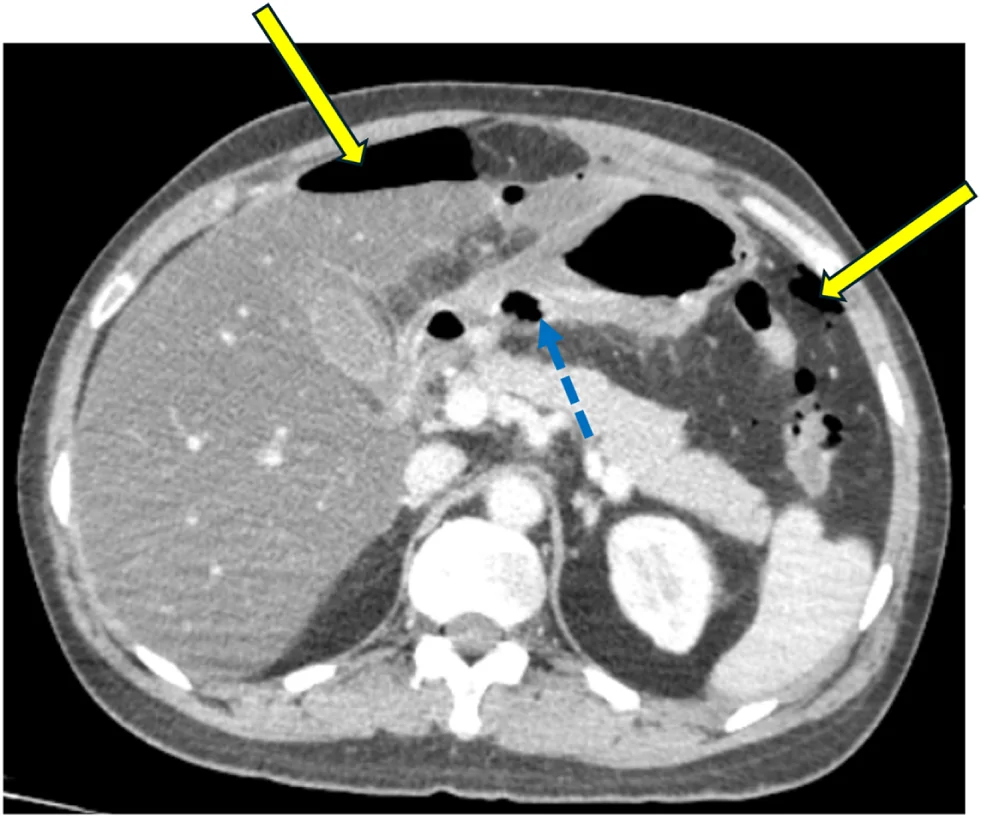
CT scan shows free air (yellow arrows) and a perforation in the stomach (blue dashed – line arrow).

**Figure 2.**
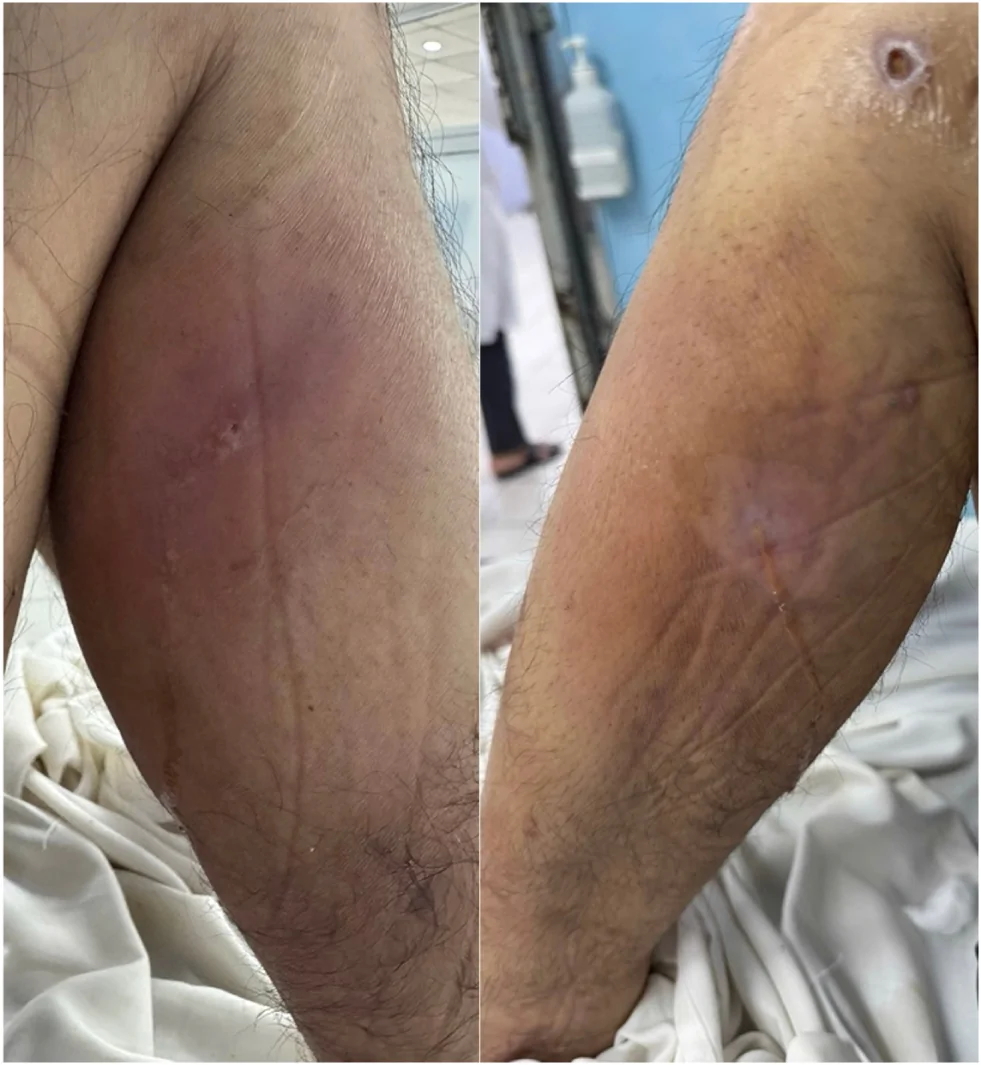
Bilateral lower-limb cellulitis.

Laboratory investigations showed mild leukocytosis, 12.5 × 10^9^/L; hemoglobin was 13 g/dL; C-reactive protein (CRP) was elevated at 40 mg/dL; the serum glucose level at admission was 5.5 mmol/L; procalcitonin testing was not performed in this case. Abdominal X-ray revealed subdiaphragmatic free gas. An abdominal computed tomography (CT) scan with contrast confirmed the presence of free air and suggested a perforated ulcer on the posterior wall of the stomach (Fig. 1). The CT scan additionally demonstrated avascular necrosis of the left femoral head, which may account for the patient’s chronic groin pain, and these findings suggested bacteremia caused by a perforated peptic ulcer. Both lower extremities exhibited extensive lesions consistent with cellulitis, accompanied by a foul odor (Fig. 2). In addition, an abscess was identified in the occipital region, and the patient was then evaluated by the orthopedic and neurosurgical teams. Subsequently, we proceeded with an exploratory laparotomy in combination with simultaneous incision and drainage of multiple abscesses.

**Figure 3.**
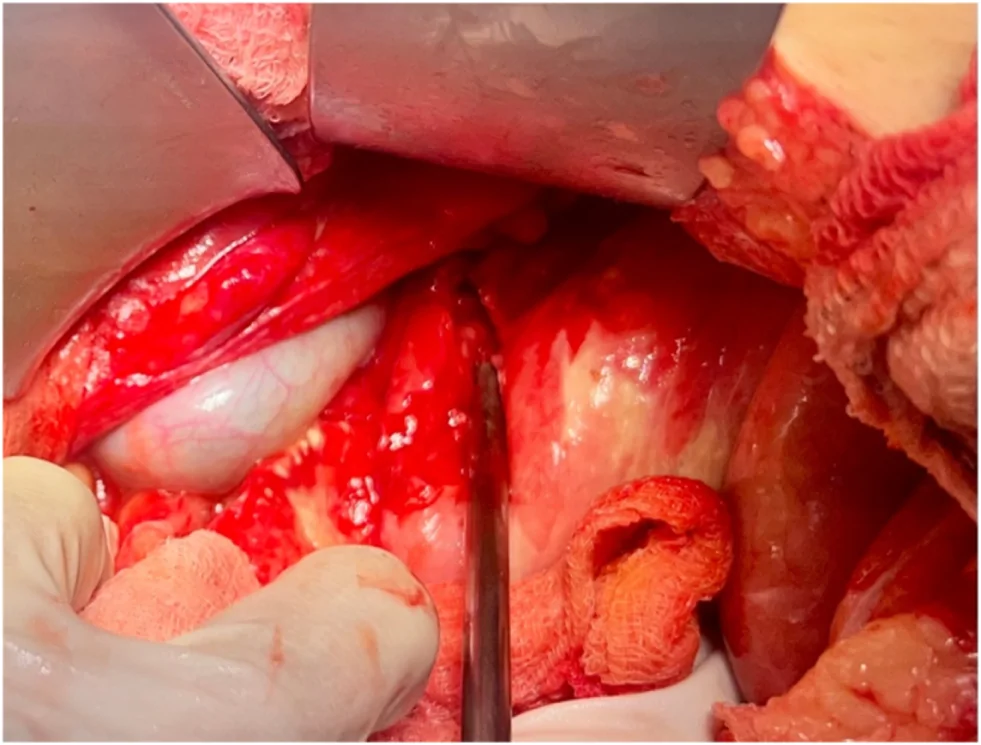
Perforated duodenal ulcer (at the tip of the suction device).

The patient was initially resuscitated with intravenous crystalloid fluids to correct dehydration, followed by intravenous antibiotic therapy guided by the hospital’s local antibiogram (imipenem/cilastatin and vancomycin), as well as acetaminophen and proton pump inhibitors (PPIs), and he was admitted to the operating room (OR) immediately. He subsequently underwent an exploratory midline laparotomy, revealing a perforated duodenal ulcer measuring 15 mm × 15 mm with well-defined, sharp margins, which suggested an acute inflammatory process rather than a neoplastic lesion, so we did not perform a biopsy. Then, the duodenal defect was closed transversely using 3-0 PDS suture (Fig. 3), and a drainage tube was placed under the liver. In Nhan dan Gia Dinh Hospital, the Graham patch is not routinely used for all cases but is more commonly performed for larger perforations (>2 cm).

Peritoneal fluid cultures yielded no growth; however, cultures from the leg and scalp abscesses isolated *Staphylococcus aureus*, which was sensitive to vancomycin. Notably, blood cultures were positive for *Salmonella* species, which was sensitive to ceftriaxone, so intravenous ceftriaxone (2 g daily) was administered for a duration of 2 weeks.

Postoperatively, the patient required admission to the intensive care unit and mechanical ventilation. An enterocutaneous fistula was noted through the drainage tube on day 8, with approximately 900 mL of fluid output; the patient’s serum albumin was low (23.3 g/L). The patient was treated conservatively with nothing by mouth and provided total parenteral nutrition (TPN). A nasogastric tube (NGT) was placed, and intermittent suction was applied at 15 mmHg. Octreotide was administered subcutaneously at a dose of 100 µg twice daily for 1 week.

The output decreased progressively over time, and after 2 weeks the leak had completely closed, and he was able to eat a soft diet. Additionally, HIV screening was performed, with a non-reactive (negative) result. The patient was discharged after 3 weeks, and the fistula was completely closed. Follow-up endoscopy was performed for *H. pylori* testing, and the result was negative; the ulcer had healed, and no evidence of malignancy was identified.

## Discussion

According to the World Society of Emergency Surgery (WSES) 2020 guidelines, peptic ulcer disease is common, with a lifetime prevalence in the general population of 5–10% and an incidence of 0.1–0.3% per year. The increasing incidence of *H. pylori* infection and the extensive use of NSAIDs have changed the epidemiology of this disease. Complications are still encountered in 10–20% of these patients^[^8^]^. In this case, the patient’s long-term use of unknown analgesics to manage chronic groin pain, later attributed to femoral head necrosis, may have contributed to ulceration. Due to the presence of generalized peritonitis and severe bacteremia, an open surgical approach was undertaken to achieve rapid source control.

Gastrointestinal leak after operative repair of perforated peptic ulcer disease (PPUD) is associated with significant post-operative morbidity. Hypoalbuminemia and duodenal perforations are significant risk factors for post-operative leaks^[^9^]^. According to Qin-Qing Tang *et al*, nutritional support plays a key role in the management and successful closure of enterocutaneous fistulas (ECF). Enteral nutrition (EN) plays an essential role in the management of ECF, supplemented by parenteral nutrition if necessary^[^10^]^. On postoperative day 8, the fistula output reached 900 mL/day, and according to Se Hwan Kwon *et al*, this would be classified as a high-output fistula^[^11^]^. We discontinued enteral nutrition temporarily, and total parenteral nutrition (TPN) was initiated; intermittent suction through the NGT was applied as well. The fistula output was closely monitored on a daily basis. Over time, the output gradually decreased, and the fistula closed completely after 2 weeks.

The simultaneous presence of soft-tissue infection further complicated management. The scalp and lower-limb abscess cultures grew Staphylococcus aureus, while peritoneal fluid cultures were negative. Waste collection and sanitation work can expose workers to diverse microorganisms through inhalation and contaminated hand contact. A study from Denmark in the occupational health field found that many people working in garbage collection were exposed to 38 different bacterial species. The genus *Micrococcus*, followed by *Staphylococcus*, constituted the largest proportions of all bacteria in both truck cab and personal samples^[^12^]^. Blood cultures were obtained prior to the initiation of empiric intravenous antibiotic therapy (imipenem/cilastatin and vancomycin). Blood culture results identified *Salmonella*, which was sensitive to ceftriaxone; we then initiated ceftriaxone for 2 weeks. *Salmonella* bloodstream infection may occur after foodborne or fecal-oral acquisition^[^13,14^]^. In this patient, the source and route of infection could not be confirmed. Possible routes include contaminated food or water exposure and environmental contact related to sanitation work; however, no direct microbiological link between the occupational environment and the patient’s cultures was demonstrated. *Salmonella bacteremia* may therefore have represented either a contributing infectious factor or a secondary bloodstream infection in the setting of severe intra-abdominal sepsis. This distinction is important because *Salmonella*-associated gastroduodenal perforation is rare, whereas enteric fever-associated perforation more commonly involves the ileum^[^6^]^; only a single case report describing this association has been identified in the literature (from India)^[^15^]^. In this patient, given the history of analgesic misuse, the perforation likely resulted from co-existing mechanisms: the chemical mucosal damage from NSAIDs combined with the systemic inflammatory stress of *Salmonella bacteremia*.

## Conclusion

This case emphasizes that *Salmonella bacteremia*, multifocal soft-tissue infection, and perforated duodenal ulcer can coexist in an otherwise healthy adult. A multidisciplinary and individualized approach is essential. Careful postoperative management, including nutritional support and close monitoring, may allow successful nonoperative closure of enteric fistulas.

## Data Availability

The data supporting the findings of this study are available from the corresponding author upon reasonable request, subject to appropriate ethical and privacy considerations.
